# Natural variation in rice ascorbate peroxidase gene *APX9* is associated with a yield-enhancing QTL cluster

**DOI:** 10.1093/jxb/erab155

**Published:** 2021-04-08

**Authors:** Yun-A Jeon, Hyun-Sook Lee, Sun-Ha Kim, Kyu-Chan Shim, Ju-Won Kang, Hyun-Jung Kim, Thomas H Tai, Sang-Nag Ahn

**Affiliations:** 1 Department of Agronomy, College of Agriculture and Life Sciences, Chungnam National University, Daejeon 34134, Republic of Korea; 2 Department of Southern Area Crop Science, Rural Development Administration, Miryang 50424, Republic of Korea; 3 LG Chem Ltd, Seoul 07796, Republic of Korea; 4 USDA-ARS Crops Pathology and Genetics Research Unit, Davis, CA 95616, USA; 5 Department of Plant Sciences, University of California, Davis, CA 95616, USA; 6 University of Birmingham, UK

**Keywords:** Ascorbate peroxidase 9, domestication, near-isogenic line, pleiotropy, rice, yield-enhancing QTL cluster

## Abstract

We previously identified a cluster of yield-related quantitative trait loci (QTLs) including plant height in CR4379, a near-isogenic line from a cross between *Oryza sativa* spp. *japonica* cultivar ‘Hwaseong’ and the wild relative *Oryza rufipogon*. Map-based cloning and transgenic approaches revealed that *APX9*, which encodes an l-ascorbate peroxidase 4, is associated with this cluster. A 3 bp InDel was observed leading to the addition of a valine in Hwaseong compared with *O. rufipogon*. *APX9-*overexpressing transgenic plants in the Hwaseong background were taller than Hwaseong. Consistent with these results, *APX9* T-DNA insertion mutants in the *japonica* cultivar Dongjin were shorter. These results confirm that *APX9* is the causal gene for the QTL cluster. Sequence analysis of *APX9* from 303 rice accessions revealed that the 3 bp InDel clearly differentiates *japonica* (*APX9*^HS^) and *O. rufipogon* (*APX9*^OR^) alleles. *indica* accessions shared both alleles, suggesting that *APX9*^HS^ was introgressed into *indica* followed by crossing. The finding that *O. rufipogon* accessions with different origins carry *APX9*^OR^ suggests that the 3 bp insertion was specifically selected in *japonica* during its domestication. Our findings demonstrate that *APX9* acts as a major regulator of plant development by controlling a valuable suite of agronomically important traits in rice.

## Teaser

DNA marker-based smart breeding revealed that the ascorbate peroxidase gene *APX9* of the wild rice relative *Oryza rufipogon* with low yield can make cultivated rice more productive and stress-tolerant.

## Introduction

Rice (*Oryza sativa* L.) is a staple for more than one-third of the world’s population. As the population continues to grow, it is necessary to increase food production to meet worldwide demand (Khush, 2003). Wild relatives of rice are a rich source of desirable genes for yield, disease resistance, stress tolerance, and other traits (Brar and Khush, 1997; Xiao *et al.*, 1998). Exploring wild and exotic rice germplasm for useful genes and transferring them into cultivars through crossing and marker-assisted selection has been shown to be feasible for increasing yields and augmenting stress resistance (Price *et al.*, 2002; McCouch *et al.*, 2007).

Rice is subjected to various abiotic and biotic stresses that lead to yield reduction. These stresses increase the production of reactive oxidative species (ROS), which in turn cause oxidation damage. Sequential reduction of molecular oxygen produces hydrogen peroxide (H_2_O_2_), superoxide radicals (O^2–^), and hydroxyl radicals (OH^•^) by electron transport systems in different subcellular compartments including the cytosol and chloroplast (Dias *et al.*, 2014). The control of intracellular ROS levels is a very complex process that involves a large network of genes, whose principal function is to avoid cellular damage that could result in oxidative stress and disturbances in cellular redox homeostasis (Mittler *et al.*, 2004). To maintain redox homeostasis, an antioxidant defense system removes free radicals and keeps the cellular steady-state level of ROS under tight control. This system consists of low-molecular-weight compounds including ascorbate and, glutathione, and enzymes such as superoxide dismutase (SOD), ascorbate peroxidase (APX), and peroxidase (POD) (Scandalios, 2002; Mittler *et al.*, 2004). APX is reported to be an efficient regulator of ROS as it contributes maximally to H_2_O_2_ detoxification, using ascorbate as an electron donor to reduce H_2_O_2_ to water (Shigeoka *et al.*, 2002).

Rice APXs are encoded by a gene family of eight members located in different subcellular compartments (Teixeira *et al.*, 2004). The expression of APXs is modulated by diverse environmental stresses (e.g. drought, salt, temperature extremes, and oxidation), and the activity of APXs against H_2_O_2_ homeostasis is important for plant stress response and growth (Agrawal *et al.*, 2003; Teixeira *et al.*, 2004; Rosa *et al.*, 2010). Roles of APX have been reported in a number of plants (Ishikawa and Shigeoka, 2008). The cytosolic *OsAPX1* rice mutant showed reduced panicle size, panicle weight, and grain yield compared with the wild type (Kim *et al.*, 2015). Similar results were found in wheat (*Triticum aestivum* L.), where the knockout of *tAPX* reduced photosynthetic activity and plant growth (Danna *et al.*, 2003). Silencing of the *OsAPX4* gene led to early senescence, suggesting a role for this gene in the senescence process in rice (Ribeiro *et al.*, 2017). In *Arabidopsis thaliana*, *AtAPX3* in the peroxisome may affect ROS accumulation caused by stress (Narendra *et al.*, 2006), and *AtAPX4* has been shown to regulate seed vigor and seedling growth (Wang *et al.*, 2014). To our knowledge, no study has analyzed the function and effect of *APX* genes on agronomic traits using genetic materials such as near-isogenic lines (NILs) in rice.

A number of quantitative trait locus (QTL) studies have shown the association of one genomic region with several traits, especially yield component traits, indicating linkage and/or pleiotropic effects (Tian *et al.*, 2006; Tan *et al.*, 2008). In some cases, the question of pleiotropy versus tight linkage has been resolved following high-density mapping and subsequent cloning of genes underlying the QTL in question. Xue *et al.* (2008) reported that the QTL *Ghd7*, encoding a CCT domain protein, has major effects on an array of traits, including the number of grains per panicle, plant height, and heading date. Wei *et al.* (2010) demonstrated that the QTL *DTH8* encodes a putative HAP3 subunit of the CCAAT-box-binding transcription factor and regulates heading date, plant height, and number of grains per panicle.

The *Oryza rufipogon* species complex is the progenitor of cultivated rice, *O. sativa*. The use of *O. rufipogon* provides an opportunity to study the morphological traits under selection during domestication. Some of the important domestication-related genes cloned in rice include *Sh4* and *qSh1* for reduction in grain shattering (Konishi *et al.*, 2006; Li *et al.*, 2006), *Rc* for red pericarp (Sweeney *et al.*, 2006), *An-1* for awn development (Luo *et al.*, 2013), and *qHD7.2* for heading date (Li *et al.*, 2018).

Previously, a single *O. rufipogon*-derived introgression on chromosome 9 in a near-isogenic line in the Hwaseong (temperate *japonica*) background was associated with agronomically important traits including grain weight and days to heading at two experimental sites located at Chungnam National University, Daejeon (Xie *et al.*, 2008) and at Chungnam Agricultural Research and Extension Services, Yesan (Yun *et al.*, 2016), both in the Republic of Korea. Here, we report that the l-ascorbate peroxidase 4 gene *APX9* (LOC_Os09g36750) is the causal gene for this QTL cluster using map-based cloning and transgenic approaches. Our data indicate that *APX9* plays an important role in the regulation of yield component traits.

## Materials and methods

### Plant material

**Fig. 1.**
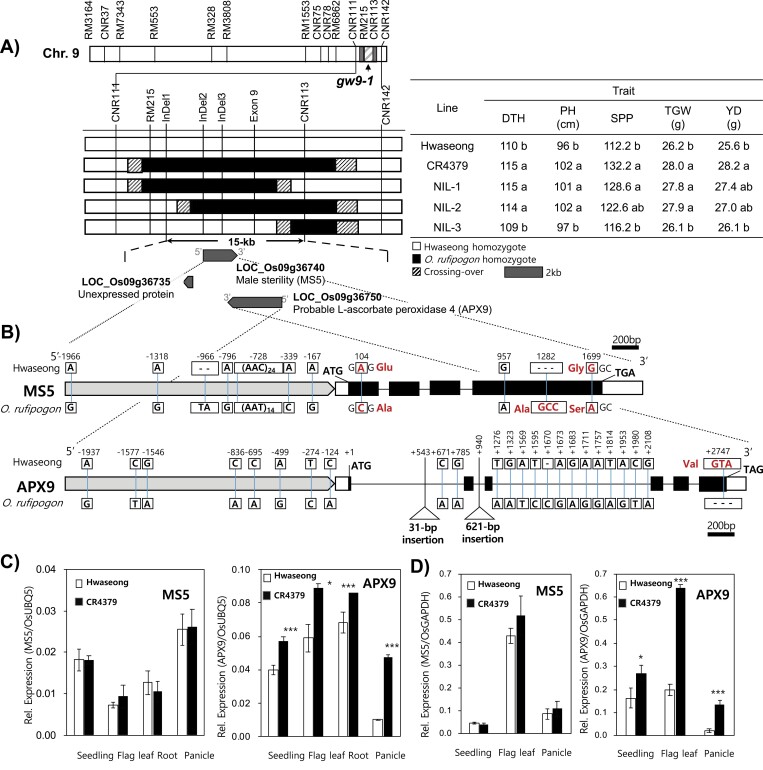
Fine mapping of the chromosome 9 QTL cluster and expression pattern of two candidate genes. (A) Substitution mapping with three BC_4_F_4_ recombinants. The white and black portions of the graphical representations of the genotypes are homozygous Hwaseong and *O. rufipogon*, respectively; the shaded gray portions indicate recombination breakpoints. The table to the right shows the mean values of DTH, PH, SPP, TGW, and YD for each line. Different letters in each column indicate significant differences among the lines (*P*<0.05; Tukey test). (B) *APX9* and *MS5* gene structure and sequence variants. Promoters are indicated by gray boxes, the white boxes indicate the 5′ and 3′ UTR regions, black boxes indicate exons, and lines between the black boxes indicate introns. Letters above and below the boxes refer to the sequences of the genes in Hwaseong and *O. rufipogon*, respectively. Non-synonymous nucleotide substitutions are indicated in red. Positions of bases are indicated by vertical lines with numbers. Bar=200 bp. (C) Expression profiles of *APX9* and *MS5* in various tissues of Hwaseong and CR4379, as determined by qRT–PCR analysis. Rice *ubiquitin5* (C) and *OsGAPDH* (D) were used as normalization controls. Error bars indicate the SD of three replicates. *P<0.05, ** *P*<0.01, ***P<0.001 (Student’s *t*-test).

In a previous study (Xie *et al.*, 2008), a cluster of yield-related QTLs was detected near RM215 on chromosome 9. CR4379 (BC_3_F_4_), harboring the target QTL from a cross between Hwaseong and *O. rufipogon* (IRGC 105491), was crossed to Hwaseong. F_1_ plants were selfed to produce an F_2_ population (>2500). These plants were screened with two simple sequence repeat (SSR) markers (RM215 and CNR113) flanking the cluster, and four recombinant BC_4_F_2_ plants were detected. As two of the four recombinants had the same recombination breakpoints, only three plants were advanced to BC_4_F_4_. Three recombinants with Hwaseong and CR4379 as controls were grown in the field and also used for substitution mapping (Fig. 1). Transgenic *APX9-*overexpressing lines (OE; T_1_ generation) were produced and grown in a greenhouse and the field with the controls. T-DNA insertion mutants in an *APX9*-insertion mutant (PFG_1B-12018.L; *japonica* ‘Dongjin’ background) and *MS5* (PFG_2A-40219R; *japonica* ‘Hwayeong’ background) were obtained from Kyunghee University, Yongin, Republic of Korea, and used for evaluating agronomic traits in T_2_ plants (Jeon *et al.*, 2000; Jeong *et al.*, 2006).

### Field trials and trait evaluation

In 2018, 29-day-old seedlings of the three recombinants, Hwaseong, and CR4379 were transplanted (one seedling per hill in three rows) in the experimental field at Chungnam National University, Daejeon. The field experiment was laid out in a completely randomized block design with two replicates. In addition, 29-day-old seedlings of *APX9-*OE and T-DNA insertion lines, with Hwaseong and Dongjin as controls, were transplanted (one seedling per hill in one row) in the experimental field at Chungnam National University. The field experiment was laid out in a completely randomized block design with one replicate. Planting distance between plants and rows was 15 cm and 30 cm, respectively. Data were recorded from 10–12 plants in the middle of each row. Data were recorded for plant height (PH), days to heading (DTH), spikelets per panicle (SPP), grain length (GL), grain width (GW), 1000-grain weight (TGW), and yield per plant (YD). Measurement of traits was performed as described in Xie *et al.* (2008).

### DNA extraction

DNA from the F_2_ population, parental lines, and transgenic lines was extracted from leaf tissues as described in Shim *et al.* (2019). PCR was conducted as described in Shim *et al.* (2019). PCR products were separated on 3% MetaPhor agarose stained with StaySafe Nucleic Acid Gel Stain (RBC, Taiwan) or 4% polyacrylamide gel stained with Silver Staining Kit (Bioneer, Daejeon, Republic of Korea).

### Analysis of transcript levels of *APX9* and *APX* genes

Total RNAs were isolated from various tissues of rice sampled at two time points in 2018 and 2019 (2-week-old seedlings, root, flag leaf, and ~5 mm-sized panicle sampled ~25 days before heading) using RNAiso Plus (TaKaRa, Shiga, Japan). Total RNA from each tissue (1 µg) was simultaneously synthesized to cDNA and amplified using a kit (SMART GENE, Daejeon, Korea). Semi-quantitative RT–PCR was performed for 25 cycles using gene-specific primer sets. PCR products were analyzed in triplicate by gel electrophoresis and band intensity was quantified using an OPTINITY Digital Gel Documentation System (GDS200, Korea Lab Tech, Seongnam, Republic of Korea). Quantitative real-time PCR (qRT–PCR) was performed using gene-specific primers in a total volume of 20 μl with 1 μl of reverse-transcription reactions as template on an CFX real-time PCR machine using SYBR Green Master mix (SMARTGENE, Daejeon, Republic of Korea). Rice *UBQ5* (*Ubiquitin5*) and *GAPDH* (*Glyceraldehyde 3-phosphate dehydrogenase*) were used as an internal reference. All experiments were conducted at least three times, with samples at each point. PCR amplifications were performed using gene-specific primers including *APX9* (Supplementary Table S1).

### Vector construction and development of transgenic lines

To produce transgenic plants overexpressing *APX9*, *O. rufipogon* cDNA was subcloned using a T-Blunt™ PCR Cloning Kit (Solgent, Daejeon, Republic of Korea). The first step was PCR amplification with the gene-specific primer sets for BamHI-*APX9*: 5′ GGGATCCCGTCAATGGTTCTTTAATAAAT-3′ RT and SacI-*APX9*: 5′-CGAGCTCGCATTTAAACTGTCATTTCATAG-3′ including restriction enzyme sites. The full-length coding region of *APX9*^OR^ (*O. rufipogon* allele) was ligated using the destination vector (*pUBI1300*; modified pCAMBIA1300) to create the final vector. The *pUBI1300* vector was equipped with the 5′ upstream region of the maize ubiquitin constitutive promoter.

For subcellular localization, the ORFs of *APX9*^HS^ (Hwaseong allele) and *APX9*^OR^ without a stop codon were amplified using gene-specific primers (forward: 5′-CGCTCGAGATGCTGATCTTTATCAGC-3′ and reverse: 5′-GCGGATCCCTTGGTTTTCTTAGAAGA-3′), which were introduced into *Xho*I- and *Bam*HI-digested *p326-GFP* plasmids using Ligation High (TOYOBO, Osaka, Japan). The resultant *p326-APX9*^HS^ and *p326-APX9*^OR^ plasmids were sequenced to confirm the absence of PCR errors. The plasmids were introduced into rice leaf protoplasts using polyethylene glycol-mediated transformation (Hayashimoto *et al.*, 1990).

**Fig. 2.**
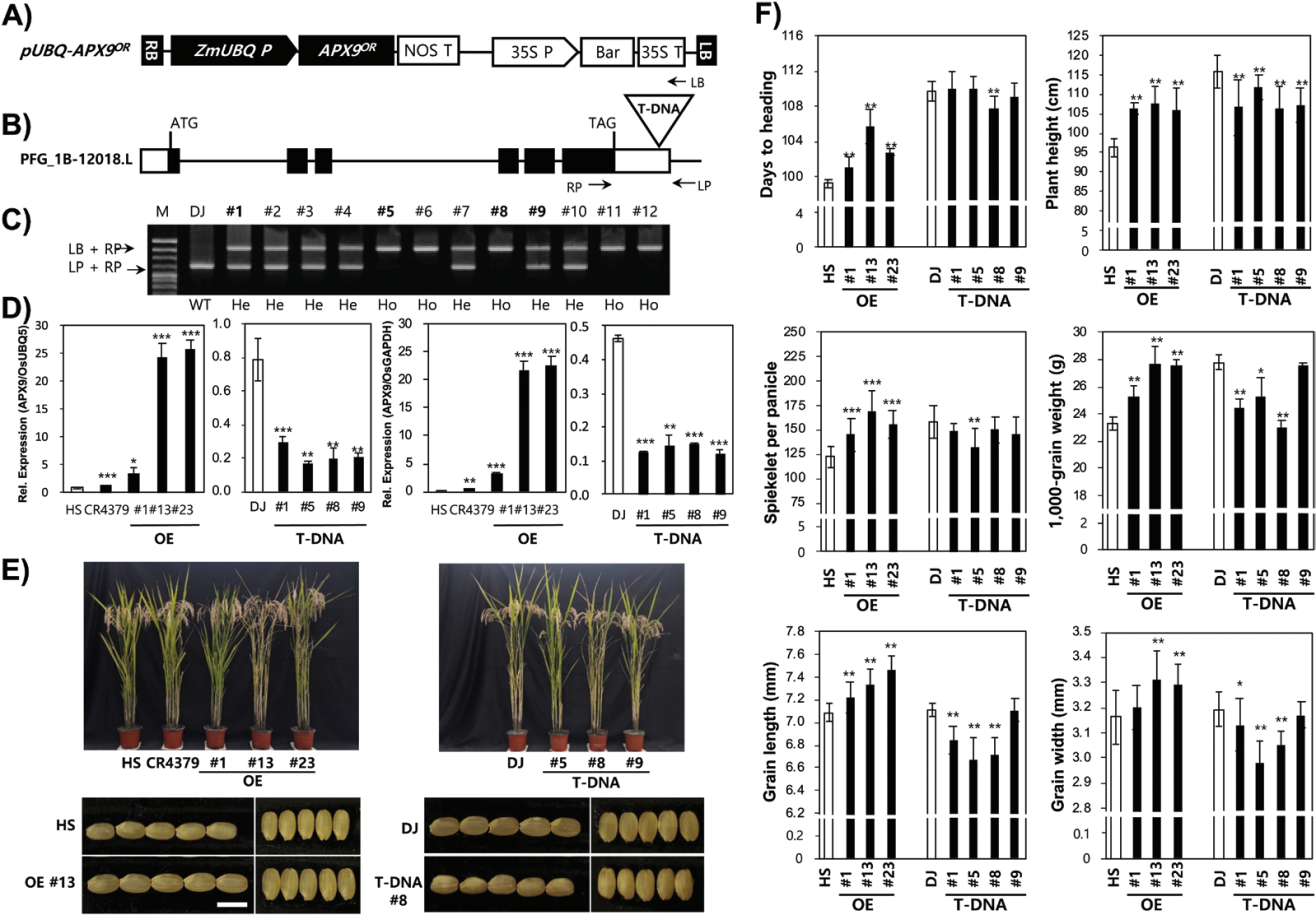
Analysis of transgenic plants and evaluation of agronomic traits. (A) Structure of the *pUBQ-APX9*^*OR*^ overexpression vector. ZmUBQ P, maize ubiquitin constitutive promoter; NOS T, nopaline synthase terminator; 35S P, cauliflower mosaic virus 35S promoter; Bar, selectable marker; 35S T, cauliflower mosaic virus 35S terminator; LB, left border; RB, right border. (B) Structure of the PFG_1B-12018.L T-DNA mutant. The white boxes indicate the 5′ and 3′ UTR regions, black boxes indicate exons, and lines between the black boxes indicate introns. (C) PCR products from Dongjin (DJ) and 12 T_1_ plants amplified with the LP and RP (gene-specific primers) or LB (T-DNA left border primer) primers. Ho, homozygote; He, heterozygote. (D) qRT–PCR analysis of expression levels in leaves of Hwaseong (HS), CR4379, and overexpressing (OE) lines, DJ, and T-DNA insertion mutants. *OsUBQ5* and *OsGAPDH* were used as controls. (E) Images showing the plant height and grain shape of Hwaseong, CR4379 and OE lines, and Dongjin and T-DNA lines. Scale bar=5 mm. (F) Comparison of DTH, PH, SPP, TGW, GL, and GW between the wild type (HS or DJ) and each transgenic line. *P<0.05, **P< 0.01, ***P<0.001 (Student’s *t*-test).

T-DNA insertion mutant lines for *APX9* and *MS5* were obtained from Kyunghee University. Mutants were grown on Murashige and Skoog medium with hygromycin (250 mg ml^–1^) to select T-DNA insertion T_1_ plants. To identify T-DNA insertion sites within the genes and select homozygous insertion lines, primers were designed for T-DNA border sequences and the flanking region of *APX9* and *MS5*. The insertion sites were amplified by PCR using left and right primers and primers specific for the left border (Fig. 2B). T_2_ seeds from each T_1_ plant were harvested and grown for further analysis.

### Rice protoplast isolation and subcellular localization of *APX9*

Approximately 100 brown rice seeds of Hwaseong were sterilized with 70% ethanol (for 2 min) followed by 50% sodium hypochlorite (for two periods of 25 min) and thoroughly washed 10 times with sterile distilled water. Protoplast isolation and polyethylene glycol-mediated transient transformation followed the methods described by Zhang *et al.* (2011) with some modifications. A fluorescence super-resolution confocal laser scanning microscope was used for fluorescence detection and image capture (LSM 880, Carl Zeiss, Germany).

### Analysis of antioxidant ability and APX activity

*In situ* detection of H_2_O_2_ was performed by staining with 3,3′-diaminobenzidine (DAB) according to the method described in Chadwick *et al.* 1995). To measure the peroxide contents, flag leaves of Hwaseong and CR4379 were soaked in DAB solution (1 mg ml^–1^ DAB with HCl, pH 3.8) for 7 h at 25 °C under continuous light. After the treatment, the leaves were washed with 90% ethanol and then heated in boiling water until the chlorophyll was completely removed.

### Abiotic stress treatments

Rice seeds were surface sterilized for 2 min with ethanol (70% v/v) and 20 min with commercially diluted (1:2 v/v) NaOCl twice, followed by rinsing 10 times with sterile distilled water. To observe the expression pattern after drought treatment, 10-day-old seedlings were air-dried for 0, 1, 2, 4, and 8 hours. The seedlings were sampled for isolation of total RNA as described above. *UBQ5* and *GAPDH* were was used as internal references. All experiments were conducted in three replicates with samples at each time point. For comparing drought tolerance among Hwaseong, CR4379, Dongjin, and the transgenic lines, water was withheld from ~15–20 10-day-old seedlings for 6 days, followed by recovery for 3 days. Phenotypic changes were photographed and the survival rates (seedlings survived after recovery/total seedlings) were calculated. Each treatment was performed in triplicate.

### Protein electrophoresis and activity staining

For the extraction of APX, 2-week-old seedlings of Hwaseong, CR4379, three OE lines, Dongjin, and four T-DNA insertion lines were homogenized in 50 mM HEPES and 0.1 mM EDTA, pH 7.0. The homogenate of each sample was transferred to a 1.5 ml tube and centrifuged at 13 475 *g* for 15 min at 4 °C. All reactions were performed on ice. The supernatant was collected and protein content was estimated according to the dye-binding method using BSA as a standard (Bradford, 1976). For the APX activity-gel assays, ~5 μg of total protein was loaded on a non-denaturing 10% polyacrylamide gel. Electrophoretic separation was performed at 4 °C for 6 h with a constant current of 15–20 mA per gel. For the analysis of APX activity, 2 mM l^–1^ ascorbic acid (AsA) was added to the electrode buffer and the gel was pre-run for 30 min before the samples were loaded (Mittler and Zilinskas, 1993). The gel was then incubated with 50 mM sodium phosphate buffer (pH 7.0) containing 4 mM AsA and 2 mM H_2_O_2_ for 20 min in the dark. The gel was subsequently washed with sodium phosphate buffer (pH 7.8), 28 mM tetramethylethylenediamine, and 2.45 mM nitroblue tetrazolium with gentle mixing for ~5 min, and the reaction was stopped by a brief wash with distilled water in the light. After staining, an achromatic band appeared against the dark purple background.

### Haplotype analysis and genotyping with InDel marker

For analysis of the APX family, the amino acid sequences of rice APXs were downloaded from the NCBI (http://www.ncbi.nlm.nih.gov/) and RAP-DB (http://rapdb.dna.affrc.go.jp) databases. A phylogenetic tree was generated with the MegAlign program in the LASERGENE package (DNASTAR, Madison, WI, USA) using the ClustalW method. The sequence information of *APX9* was used to determine the phylogenetic relationships and construct haplotypes among 246 Asian cultivated rice (*O. sativa* L.) accessions from the KRICE_CORE set (Kim *et al.*, 2016); the accessions included 193 *japonica*, 48 *indica*, and 5 *aus* accessions (Supplementary Table S2). The maximum likelihood method of the MEGA program was employed to construct the phylogenetic tree (Kumar *et al.*, 2016).

To determine the distribution of the 3 bp insertion/deletion (InDel), PCR analysis was performed to detect the presence of the InDel in 145 accessions (Supplementary Table S2). These accessions included 73 *japonica* (56 temperate and 17 tropical *japonica*), 41 *indica*, 3 *admixture*, 2 *aromatic*, 6 *aus*, and 20 wild rice including *O. rufipogon*. Forward and reverse primers for the 3 bp InDel marker are shown in Supplementary Table S1.

### Genetic diversity analysis

Genomic sequences of 41 genes of 86 accessions (Supplementary Table S3) encompassing an 820 kb region upstream and downstream of *APX9* were downloaded from the TASUKE database and used for population genetic analysis using DnaSP5.1 (Librado and Rozas, 2009). Levels of nucleotide diversity per site and silent site were estimated as a π value for each group.

### Statistical analysis

QTLs were fine mapped by comparing the phenotypic means of genotypic classes of recombinants within the target region using the ANOVA feature in Minitab 19; values of *P*<0.05 were considered to be significant. The observed phenotypic variation was estimated using the coefficient of determination. For Tukey’s test, Minitab19 software was used. Student’s *t*-test was conducted using Microsoft Excel.

## Results

### Substitution mapping of the QTL cluster and analysis of candidate genes

Four BC_4_F_2_ recombinants between the markers CNR111 and CNR142 were identified by screening over 2500 plants from a cross between the NIL CR4379 (BC_3_F_4_) and Hwaseong. These recombinants were advanced to obtain a BC_4_F_4_ generation and three of the four BC_4_F_4_ NILs were genotyped with six additional markers and were found to be fixed for Hwaseong or *O. rufipogon* segments (Fig. 1A). NIL-1 plants had *O. rufipogon* segments between CNR111 and CNR113, whereas NIL-2 and NIL-3 had *O. rufipogon* segments between InDel1 and CNR142, and between exon 9 and CNR142, respectively (Supplementary Table S1). Five agronomic traits were evaluated: DTH, PH, SPP, TGW, and YD. Significant differences between Hwaseong and two NILs (NIL-1 and NIL-2) were observed for all the traits (Fig. 1A). In contrast, Hwaseong and NIL-3 plants did not show differences in any of the traits. These results indicate that the QTL cluster resides between InDel1 and CNR113, a region of ~14.9 kb.

Sequence annotation databases (https://rapdb.dna.affrc.go.jp/index.html) revealed three predicted genes in the target region, including an unexpressed protein (LOC_Os09g36735), a male sterility gene (LOC_Os09g03640; *Male sterility 5*, *MS5* hereafter), and a probable l-ascorbate peroxidase 4 (LOC_Os09g36750, *APX9* hereafter) (Fig. 1A). The unexpressed protein was considered an unlikely candidate and excluded from further analysis. Sequencing of the region spanning the *APX9* and *MS5* genes from Hwaseong and *O. rufipogon* was performed to look for possible functional polymorphisms. For *APX9*, numerous single nucleotide polymorphisms (SNPs) were identified in the promoter region (upstream 2 kb) and introns (Fig. 1B). Two insertions, 31 bp and 621 bp in length, were found in the first and second intron, respectively, of the *O. rufipogon* (*APX9*^OR^) allele. In addition, a 3 bp difference was identified near the stop codon in the sixth exon. This InDel resulted in the addition of a valine to the protein encoded by the Hwaseong allele (*APX9*^HS^). Sequencing of the *MS5* region revealed five SNPs and two InDels in the promoter and 5′ UTR regions (Fig. 1B). One SNP in the first exon of *MS5* is predicted to encode a glutamine in Hwaseong and an alanine in *O. rufipogon*. In the last exon, three polymorphisms were detected, including SNPs at position 957 (no predicted effect) and 1699 (glycine in Hwaseong and serine in *O. rufipogon*), and a 3 bp InDel resulting in an additional alanine in the predicted *O. rufipogon MS5* protein (Fig. 1B).

Gene expression analysis using qRT–PCR was conducted on 2-week-old seedlings, flag leaf, young panicle, and roots (Supplementary Table S1). Values were calculated based on the expression of the target gene relative to the expression of *OsUBQ5* and *OsGAPDH* (Fig. 1C, D). The expression level of *MS5* in Hwaseong and CR4379 was similar, whereas CR4379 plants showed higher expression of *APX9* than Hwaseong in all tissues examined over 2 years. The largest fold difference in expression of *APX9* in CR4379 compared with Hwaseong was observed in young panicle, with a 4.6-fold increase. These results suggest that *APX9* is most likely responsible for the QTL cluster.

### Functional analysis of *APX9*

Transgenic plants overexpressing *APX9* in the Hwaseong background were generated, and insertion mutant lines derived from the cultivar Dongjin and harboring T-DNA insertions in the 3′ UTR region were obtained (Fig. 2). A vector overexpressing *APX9*^OR^ using the maize ubiquitin constitutive promoter was constructed and transformed into Hwaseong (Fig. 2A). Three OE lines (#1, #13, and #23) were selected by qRT–PCR and grown in the field for analysis (Fig. 2D). To determine the genotype of the T-DNA plants, 12 individuals were analyzed using gene-specific primers flanking the T-DNA insertion and primers to the T-DNA left border to distinguish homozygotes and heterozygotes (Fig. 2B, C). Four lines showing lower expression of *APX9* (#1, #5, #8, and #9) were selected by qRT–PCR (Fig. 2D).

The agronomic traits of the OE and T-DNA insertion lines were evaluated (Fig. 2E, F). Yield-related traits in Hwaseong, CR4379, and the transgenic lines were compared at maturity (Supplementary Table S4). The difference in DTH, PH, and SPP between all three OE lines and Hwaseong was significant (Fig. 2F). All OE lines had larger grains than Hwaseong, mainly due to increased GL and GW (Fig. 2E, F). The four T-DNA insertion lines showed variation in all traits measured. Insertion line #8 flowered earlier, and showed reduced PH and grain size compared with wild-type Dongjin, and line #5 was also shorter with fewer SPP and had smaller grains than Dongjin (Fig. 2F). Line #1 showed significant differences from Dongjin in PH, grain weight, and grain shape traits, and line #9 displayed a significant difference only for PH. 

We also evaluated *MS5* T-DNA insertion mutants derived from the cultivar Hwayeong. No consistent differences in PH and DTH were observed between T-DNA insertion mutants and wild-type Hwayeong. However, the *MS5* mutants all exhibited high spikelet sterility, which was consistent with a role of *MS5* in grain fertility (Supplementary Table S5). These results indicate that *APX9* is responsible for the QTL cluster that affects DTH, PH, SPP, GL, and GW. The performance of OE and T-DNA insertion lines suggests that the 3 bp InDel in *APX9* is associated with the QTL cluster. However, the possibility that SNPs in the promoter control the variation of the QTL cannot be ruled out.

### APX9 proteins are mainly localized in the chloroplast

**Fig. 3.**
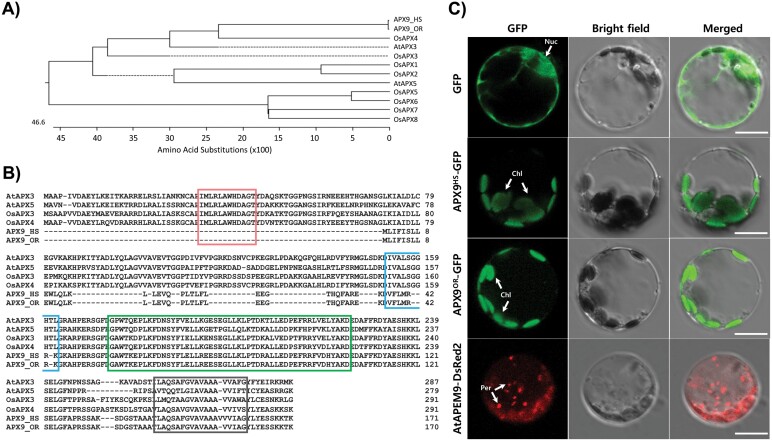
Analysis of amino acid sequence and subcellular localization of *APX9*. (A) Phylogenetic analysis of APX proteins in rice. The phylogenetic tree was constructed using ClustalW. (B) Alignment of peroxisomal APXs in Arabidopsis and rice. Active and heme-binding sites are indicated by the red and blue boxes, respectively. The green box indicates the APX family peroxidase motif. The black box indicates a transmembrane domain where the C-terminal peroxisomal targeting sequence is located. (C) Subcellular localization of the APX9^HS^ and APX9^OR^ proteins by GFP assays. 35S-*APX9*^HS^-GFP and 35S-*APX9*^OR^-GFP plasmids were transformed into rice protoplasts by polyethylene glycol-mediated infiltration. Fluorescence signals were visualized using confocal laser scanning microscopy. 35S-AtAPEM9-DsRed2, red fluorescence signals from the peroxisome (Per) marker; Merged, merged images of GFP fluorescence and bright field; Chl, chloroplast; Nuc, nucleus. Scale bar=10 μm.

A phylogenetic tree was constructed to compare the amino acid sequences of proteins encoded by *APX9* and the *APX* family (Fig. 3A). A total of eight *APX* genes are located in various organelles in rice (Teixeira *et al.*, 2006), with *APX9* being in the same group as *OsAPX3* and *OsAPX4*. The peroxisomal APX family has a C-terminal transmembrane domain for targeting to the peroxisome in various crops (Bunkelmann and Trelease, 1996). *APX9*^HS^ and *APX9*^OR^ encode proteins of 171 and 170 amino acids, respectively. *APX9* shares a high sequence homology with Arabidopsis At4g35000 (*AtAPX3*, 52% identity) and rice LOC_Os08g43560 (*OsAPX4*, 57% identity). Amino acid sequences of the Arabidopsis and rice peroxisomal APXs were aligned (Fig. 3B). All five APXs had common peroxidase motifs and peroxisomal targeting sequences. However, in APX9 the active site is absent and the heme-binding site is incomplete. *APX9*^OR^ showed a 3 bp difference in the peroxisomal targeting sequence compared with *APX9*^HS^. The putative subcellular localization predicted by the LOCALIZER, CELLO, and Plant-mPLoc databases also supports that *APX9* probably encodes a peroxisomal APX.

The APX9 protein was expressed in rice leaf protoplasts as a fusion with GFP under the control of the cauliflower mosaic virus 35S promoter, with the 35S-AtAPEM9-DsRed2 fusion protein, which targets the peroxisome, as a positive control. As shown in Fig. 3C, the fluorescence of the control GFP protein was distributed throughout the cell. Confocal microscopy of protoplasts expressing 35S-*APX9*^HS^-GFP and 35S-*APX9*^OR^-GFP revealed that the GFP was mostly localized in chloroplasts, even though APX9 has a targeting sequence for the peroxisome membrane. This result suggests that the 3 bp InDel polymorphism between the *APX9* sequences of Hwaseong and *O. rufipogon* does not affect the subcellular localization of the APX9 protein.

### CR4379 shows higher antioxidant ability and APX activity than Hwaseong

APXs are known to catalyze the reduction of H_2_O_2_ to H_2_O and O_2_ using AsA as a specific electron donor, and are the most important H_2_O_2_-eliminating enzymes in the chloroplast (Asada, 1999; Shigeoka *et al.*, 2002). To investigate whether *APX9* is involved in ROS-scavenging metabolism, we used DAB staining, which reacts with H_2_O_2_*in situ* and produces dark-brown spots (Guan and Scandalios, 2000; Zhang *et al.*, 2009). Leaves of CR4379 plants showed less pronounced DAB staining than leaves of Hwaseong, suggesting that CR4379 contained less H_2_O_2_ than Hwaseong (Supplementary Fig. S1A).

To identify enzyme activity in transgenic plants, APX activities were measured. CR4379 showed a higher level of APX activity than Hwaseong (Supplementary Fig. S1B). The OE lines #1, #13, and #23 showed higher enzyme activity than Hwaseong (Supplementary Fig. S1C), whereas the four T-DNA insertion lines were not significantly different from the wild type Dongjin (Supplementary Fig. S1D). These findings suggest that *APX9*^OR^ is more efficient than *APX9*^HS^ in regulating APX activity induction in rice.

### Performance of CR4379 and transgenic plants under drought

**Fig. 4.**
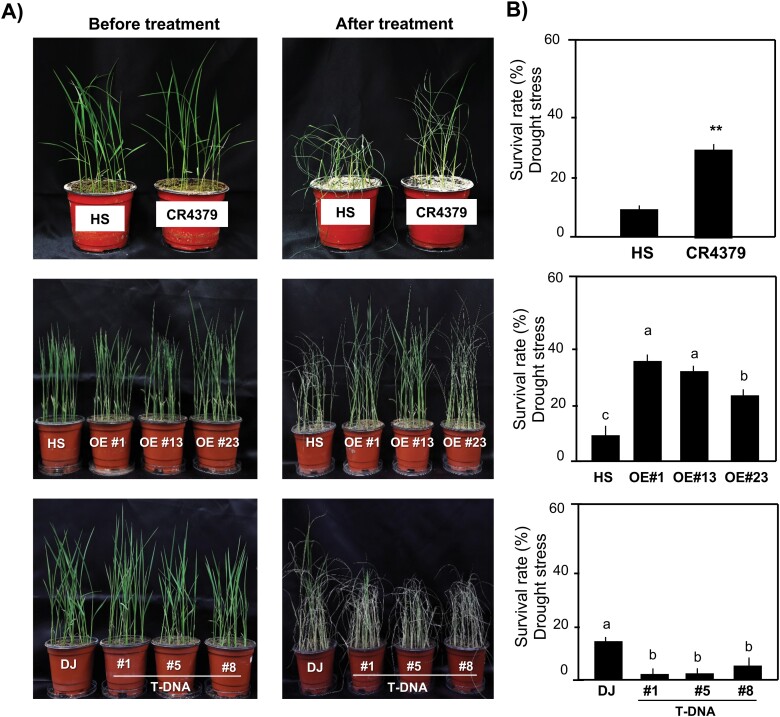
Expression levels of *APX9* under drought. (A) Representative images of wild-type and transgenic lines before and after 6 days of drought treatment. (B) Survival rates (%) of wild-type and transgenic lines after drought treatment. Ten-day-old seedlings were subjected to drought treatment for 6 days, followed by a 3-day recovery period. Each treatment involved at least 15 plants, and independent experiments were repeated more than three times. Data represent the mean ±SE calculated from three replicates. In the upper panel, a significant difference between Hwaseong (HS) and CR4379 is indicated with asterisks: **P<0.01 (Student’s *t*-test). In the middle and lower panels, significant differences between the wild-type [HS and Dongjin (DJ)] and transgenic lines are indicated with different letters above the bars (*P*<0.05; Tukey’s test). DJ T-DNA insertion line #9 was not tested due to lack of seeds.

It has been generally reported that APX plays a role in plant growth under abiotic stress conditions. Therefore, we examined the performance of Hwaseong, CR4379, and transgenic lines along with their respective wild-type plants. Seedlings of CR4379 showed enhanced drought tolerance. The survival rate of 28.9% in CR4379 was higher than that of Hwaseong (9.8%) after drought treatment (Fig. 4A, B). We also tested the OE and T-DNA insertion lines for drought tolerance and found significant differences in survival rates between the OE lines and Hwaseong. Dongjin seedlings were also more drought tolerant than the three T-DNA lines examined (Fig. 4A, B). These results suggest that overexpression of *APX9*^OR^ may have a positive effect on drought tolerance.

Under drought conditions, the expression levels of *APX9* relative to *OsUBQ5* in Hwaseong increased 1 h after treatment, whereas CR4379 displayed a gradual increase in *APX9* expression over time, reaching a peak at 8 h after treatment (1.7- to 2.6-fold higher than Hwaseong') (Supplementary Fig. S2A). Three OE lines showed different expression patterns (Supplementary Fig. S2B). The OE line #1 exhibited an approximately 2.7-fold increase in *APX9* expression relative to Hwaseong at 1 h after drought treatment, but expression decreased rapidly from 2 h after treatment. In contrast, the OE lines #13 and #23 had higher *APX9* expression levels than Hwaseong at each time point. *APX9* expression levels in Dongjin and the three T-DNA lines (#1, #5, and #8) were compared (Supplementary Fig. S2C). In Dongjin, *APX9* expression increased over time, reaching a peak at 8 h after drought treatment. The three T-DNA lines showed different expression patterns, with expression levels mostly lower than those of Dongjin over the time points examined. Similar expression patterns were observed for *APX9*/*OsGAPDH* as for *APX9*/*OsUBQ5*, except for higher expression levels in CR4379 than Hwaseong at all time points (Supplementary Fig. S2D–F). The greater drought tolerance of CR4379 and OE lines compared with Hwaseong implies that *APX9* is involved in drought tolerance.

### Expression of APX protein and enzyme activity in transgenic plants

To further investigate APX activity in CR4379 and OE lines, native gel analysis was performed on protein extracts from 2-week-old seedlings (Supplementary Fig. S3). CR4379 and three OE lines showed stronger, more intensely stained bands (~1.5–1.6-fold) than Hwaseong at the expected molecular weight range of ~25–35 kDa. Band intensities in the ~25–35 kDa range for the four T-DNA insertion lines were similar to those of their corresponding wild type, Dongjin. Since native gel analysis reflects the activity of all APX proteins, the expression of the other eight *APX* genes was analyzed to clarify the contribution of *APX9* to the observed activity. Three OE lines showed the same or similar expression levels for all eight genes compared with Hwaseong (Supplementary Fig. S4). Given that CR4379 has a single *O. rufipogon* introgression on chromosome 9 harboring *APX9*^OR^ in the Hwaseong genetic background, the difference in the APX protein activity between Hwaseong and CR4379 appears attributable to *APX9*^OR^. Even though some APX activity was lost because the extraction buffer for ascorbate peroxidase did not contain ascorbate to protect the chloroplast forms from inactivation, this would presumably have occurred in samples from both Hwaseong and CR4379. Each T-DNA insertion line showed varying expression levels of the eight other *APX* genes in comparison to their wild type, Dongjin (Supplementary Fig. S5). This may reflect background effects of T-DNA insertions in the mutant lines.

### Distribution of the InDel in *APX9* among rice accessions

To examine the distribution of the 3 bp InDel found in *APX9* and associated with the yield-enhancing QTL cluster, 303 rice accessions were selected from the KRICE_CORE set (268 accessions) and a small laboratory collection (35 accessions) for genotyping (Supplementary Table S2, Supplementary Fig. S6). The accessions consisted of 2 admixture, 3 *aromatic*, 8 *aus*, 65 *indica*, 205 *japonica* (comprising 184 temperate *japonica* and 21 tropical *japonica*), and 20 wild rice, including *O. rufipogon*. The *APX*^OR^ allele was not observed in admixture, temperate *japonica*, or tropical *japonica* accessions, and was mainly found in *indica* and *O. rufipogon*. Among the 71 accessions with the *APX*^OR^ allele, 48 (67.6%) were *indica* and 12 (16.9%) were *O. rufipogon*. In addition to all the *japonica* accessions, 17 *indica* accessions also had the *APX*^HS^ allele. The finding that most of the wild rice accessions had the *APX*^OR^ allele and all *japonica* accession had the *APX*^HS^ allele (i.e. a 3 bp insertion) suggests that the insertion occurred in *japonica* before differentiation into the tropical and temperate groups, and that the *APX*^HS^ allele was introgressed into some *indica* by crossing.

### *APX9* haplotype analysis

**Fig. 5.**
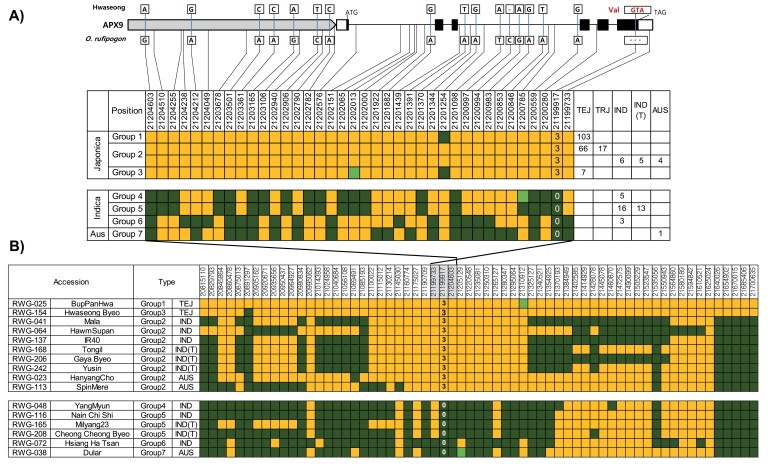
Haplotype analysis across the *APX9* gene region. (A) Sequence differences of *APX9*, comprising six exons (black boxes), 5′ and 3′ UTR (white boxes), and the promoter (gray box) across ~5 kb of genomic DNA, are shown horizontally along the top; the position of the 3 bp insertion mutation is shown in red; and SNP positions within *APX9* are connected by lines to the haplotype table below. Seven haplotypes are constructed based on the sequence variations among 246 accessions. The number of accessions in each haplotype group are indicated to the right. Yellow indicates the reference (Nipponbare) sequence, dark green indicates SNPs or InDel, and light green shows the presence of a third allele or missing data. (B) Extended haplotype table corresponding to the 885 kb region flanking the *APX9* gene. The position of the 3 bp insertion mutation within the extended haplotype is marked by the numbers 3 or 0. The colors are the same as described for (A). The nucleotide positions of 60 selected SNPs with an average interval of 15 kb along the 885 kb region in 16 accessions are shown at the top.

Haplotype analysis was performed on 246 accessions from the KRICE_CORE set to compare the sequence variation in *APX9*. Using the 38 SNPs/InDel identified in the upstream 2 kb promoter and 3 kb coding region, seven haplotype groups (HGs) were constructed (Fig. 5A). All *japonica* accessions were classified into three groups (HG1, HG2, and HG3), whereas 37 *indica* accessions formed HG4, HG5, and HG6. HG7 comprised a single *aus* accession. In the promoter, three SNPs at 21 204 603, 21 203 501, and 21 203 165 were informative in distinguishing *japonica* (HG1–3) and *indica* plus *aus* (HG4–7) accessions. In the coding region, HG1–3 was differentiated from HG4–7 at three SNPs at 21 201 098, 21 200 559, and 21 199 917 (the 3 bp InDel). Interestingly, 15 *indica* accessions (including 5 Tongil-type and 4 *aus*) were classified into HG2 with *japonica*. These results confirm that the segment harboring the *APX9*^HS^ allele was introgressed into these *indica* accessions in HG2 from *japonica* by crossing, not the other way around. To determine the size of this introgression, we examined the haplotypes of 16 of the 246 accessions across an 885 kb genomic region flanking *APX9* (Fig. 5B). These accessions were chosen from each haplotype group and included one *japonica* accession in HG1, eight (six *indica* and two *aus*) accessions in HG2, six (five *indica* and one *aus*) accessions in HG4–7, and Hwaseong as the control. The accessions were compared using 60 SNPs randomly selected along the 885 kb region (15 kb average interval). Eight accessions in HG2, harboring the same *APX9*^HS^ allele as HG1 and HG2, shared the same SNPs with two *japonica* accessions (Bup Pan Hwa and Hwaseong) in the genomic region from around nucleotide 21 100 022 to 21 310 912, indicating that these eight *indica* accessions have a *japonica* introgression of approximately 210 kb. For IR40, an *indica* accession derived from the pedigree IR20*2/*Oryza nivara*//CR94-13, the origin of its *APX9*^HS^ allele is not clear. However, the sharing of the *APX*^HS^ allele of IR40 with Gaya Byeo may be explained by the fact that one of the parents of Gaya Byeo is IR32, a sister line of IR40. Based on the haplotypes, the *japonica* introgression in IR40, Mala, and Gaya Byeo is also approximately 210 kb. Five accessions in HG4–6 harboring the *APX9*^OS^ allele also shared the same SNPs in the region upstream of nucleotide 21 354 925. All *indica* and *aus* accessions had the same SNPs, which differed from those of *japonica* in the region upstream of nucleotide 21 640 026. Two Tongil-type accessions (Milyang23 and Cheong Cheong Byeo) shared the same haplotype in the region including the *APX*^OR^ allele as the other *indica* accessions classified into HG4–5. The *APX*^OR^ allele in the two Tongil-type rice accessions, which are derived from *indica/japonica* crosses, appears to be from the crossing parent IR24, suggesting that the *japonica* segment harboring the *APX*^HS^ allele was not selected in the breeding program. The finding that only 5 of 18 Tongil-type accessions carried the *APX*^HS^ allele suggests that *APX9*^OR^ may not have been a major target trait in the *indica* breeding program.

### Genetic diversity analysis around the *APX9* region

**Fig. 6.**
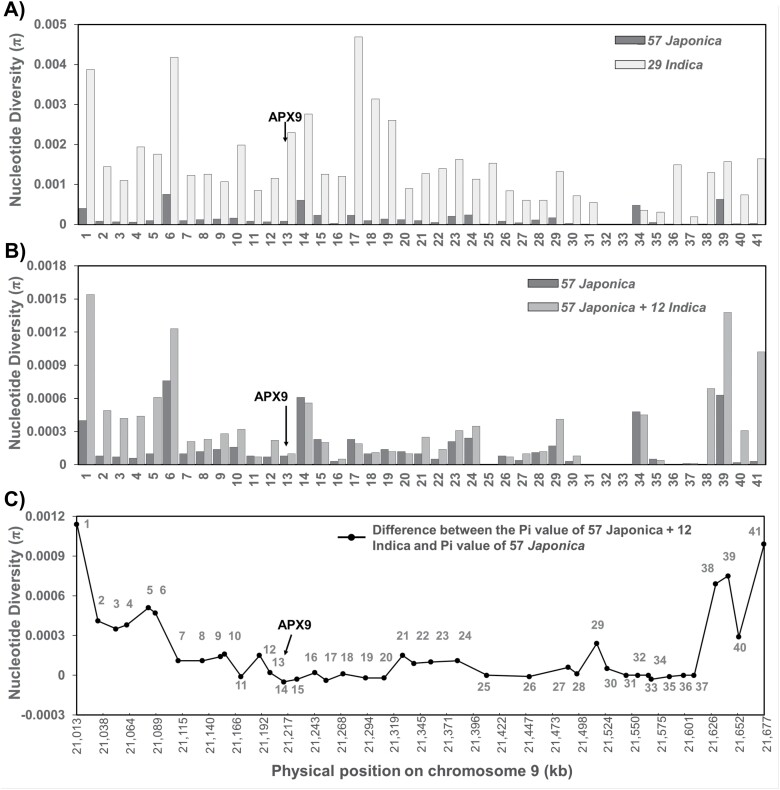
Genetic diversity analysis across *APX9* genomic regions. (A) Nucleotide diversity (π) of 57 *japonica* and 29 *indica* (comprising 24 *indica* and 5 *aus*) accessions at 41 loci, including *APX9* (no. 13). (B) π of 57 *japonica* accessions and 69 accessions (57 *japonica* plus 12 *indica* harboring the *APX*^HS^ allele) at 41 loci. (C) Difference in π between the 69 accessions and the 57 *japonica* accessions. The difference in value at each locus is due to the diversity of the 12 *indica* accessions harboring the *APX*^HS^ allele. The sequences of 41 genes in 86 rice accessions from RAP-DB and the TASUKE rice genome browser were obtained and analyzed.

To confirm the introgression of the *APX9*^HS^ genomic region from *japonica* into diverse *indica* accessions, sequences of 86 rice accessions from RAP-DB and the TASUKE rice genome browser (https://tasuke.dna.affrc.go.jp) were obtained and assayed. These accessions included 57 *japonica* (31 temperate *japonica* and 26 tropical *japonica*), 24 *indica*, and 5 *aus* (Supplementary Table S3). The nucleotide diversity (*π*) between 12 *indica* and 57 *japonica* accessions was compared to confirm the introgression event. The decline in the nucleotide diversity value between two groups in genomic regions is consistent with an introgression from *japonica*. The nucleotide diversity of *APX9* in *japonica* (*π*=0.00008) was markedly lower than that in *indica* (*π*=0.0023) (Fig. 6A). Examination of 41 genes randomly selected at intervals of approximately 16.8 kb along the 665 kb region flanking *APX9* revealed that the nucleotide diversity in *indica* (~0–0.00469) was higher than or similar to *japonica* (~0–0.00076) (Supplementary Table S6). The difference in *π* between the average values of 57 *japonica* and 69 (57 *japonica* plus 12 *indica*) accessions was calculated for the 41 genes. Interestingly, the difference was <0.0003 in the ~499 kb region from Gene7 (Os09g0536700) to Gene37 (Os09g0547200) and showed a ~2–3-fold increase from the region upstream of Gene7 and downstream of Gene38 (Fig. 6B, C). The region (21 110 946–21 613 089 bp) also overlapped with the 210 kb (21 100 022–21 310 912 bp) segment shown in Fig. 5B. These results suggest that the high diversity in *indica* is mainly due to the inclusion of *indica* accessions harboring *japonica* alleles such as *APX9*^HS^, and also that the approximately 499 kb *japonica* segment was introgressed into *indica* by crossing.

## Discussion

In this study, we demonstrated that *APX9* is associated with the QTL cluster for yield-related traits in rice. We developed BC_4_F_4_ NILs from an interspecific cross between Hwaseong and *O. rufipogon* and observed significant differences in yield-related traits between Hwaseong and NILs. Map-based cloning enabled us to delimit the QTL to a ~15 kb region flanked by the SSR markers InDel1 and CNR113 (Fig. 1A). The region contains three putative genes; an unexpressed protein (LOC_Os09g36735), *MS5* (LOC_Os09g36740), and *APX9* (LOC_Os09g36750). *MS5* is a tetratricopeptide repeat domain-containing protein and *APX9* is a putative peroxisome-type ascorbate peroxidase. To identify the causal gene(s) for the cluster, gene sequencing, expression analysis, and transgenic approaches were employed. Sequence comparison of *APX9* between the two parental lines showed the presence of the 3 bp InDel in the sixth exon, whereas two missense SNPs and an InDel were detected in *MS5* (Fig. 1B). Gene expression analysis indicated that CR4379 plants showed higher *APX9* expression in various tissues than Hwaseong plants, whereas no difference in *MS5* expression was observed between Hwaseong and CR4379. To investigate whether the cluster is controlled by tightly linked genes or a single pleiotropic gene, we generated *APX9* OE transgenic lines in the Hwaseong background and also characterized *APX9* T-DNA insertion mutants in Dongjin, another wild-type background. OE plants were taller, had heavier grains, and flowered later than Hwaseong plants, whereas the T-DNA mutants showed reduced PH and TGW compared with Dongjin (Fig. 2E, F). However, *MS5* T-DNA plants did not show differences in PH or DTH but did exhibit high sterility. Studies have reported that *APX* genes play an important role in regulating PH and plant development (Lazzarotto *et al.*, 2011; Zhang *et al.*, 2013), DTH (Miller *et al.* 2007, Chai *et al.*, 2012), panicle size and weight, and grain yield (Kim *et al.*, 2015). Together, our findings indicate that *APX9* is the causal gene for the QTL cluster.

Eight *APX* genes in rice (Agrawal *et al.*, 2003; Teixeira *et al.*, 2004) are distributed as isoenzymes in distinct cellular compartments. Based on the phylogenetic tree of the *APX* gene family in rice, *APX9* is similar to *OsAPX4* and has a predicted amino acid sequence for targeting to the peroxisome. It is interesting to note that *OsAPX4*, together with *AtAPX3* and *AtAPX5*, have active and heme-binding sites that are characteristic of ascorbate peroxidase (Teixeira *et al.*, 2004), whereas *APX9* has only the peroxidase motif and the conserved domain (Fig. 3B). This suggests that *APX9* probably reacts with different substrates from those of *AtAPX3*, *AtAPX5*, and *OsAPX4*. However, it is possible that *APX9* reacts differently to the same substrates. Another interesting point is that the 3 bp insertion in *APX9*^HS^ results in the insertion of valine in the peroxisomal targeting sequence. The cotton pAPX is localized to the peroxisomal membrane (Mullen *et al.*, 1999) and the sequence targeting it to this membrane consists of a C-terminal transmembrane domain followed by a few basic amino acid residues defined as the mPTS (i.e. the targeting signal of peroxisomal membrane-bound proteins) (Mullen *et al.*, 2000, 2001). Since the predicted C-terminal sequence encoded by *APX9* resembles the mPTS of the cotton pAPX, APX9 was predicted to be bound to peroxisomal membranes; however, our GPF localization results indicated that APX9 is mainly targeted to the chloroplast.

Whether QTLs that affect different traits and map to the same genomic region are attributable to a pleiotropic gene or the tight linkage of multiple genes that individually influence specific traits has been a topic of debate. A QTL cluster for yield-related traits has been mapped to chromosome 9 in rice, with the beneficial alleles being contributed by the wild species *O. rufipogon*. Our identification of a single gene, *APX9*, affecting the yield-related traits of this QTL cluster is consistent with previous findings of pleiotropic genes underlying QTL clusters in cereals (Xue *et al.*, 2008; Wei *et al.*, 2010; Sreenivasulu and Schnurbusch, 2011; Chen *et al.*, 2020). *Grain number, plant height, and heading date7* (*Ghd7*), encoding a CCT (CONSTANS, CONSTANS-LIKE, and TIMING OF CHLOROPHYLL A/B BINDING) domain protein, is a key regulator of the rice-specific flowering pathway and also contributes to rice yield potential (Xue *et al.*, 2008). Wei *et al.* (2010) demonstrated that a QTL, *DTH8*, encoding a putative HAP3 subunit of the CCAAT-box-binding transcription factor regulates DTH, PH, and number of grains per panicle. Chen *et al.* (2020) identified three pleiotropic QTL regions associated with spikelet number and heading date in common wheat.

Growing evidence supports that genes in the heading date pathway also affect plant development and stress response (Xue *et al.*, 2008). In this study, CR4379 plants flowered 5–6 days later under field conditions than Hwaseong. This delay is possibly due to differences in endogenous H_2_O_2_ content between the two lines. APX is an efficient regulator of ROS as it contributes maximally to H_2_O_2_ detoxification (Shigeoka *et al.*, 2002). This is consistent with previous findings that H_2_O_2_ is involved in physiological processes including development and flowering, and that the *APX* gene regulates H_2_O_2_ as well as flowering time in Arabidopsis (Chai *et al.*, 2012; Liu *et al.*, 2013). Transgenic Arabidopsis plants overexpressing *APX* showed delayed flowering compared with the wild type, whereas *APX* knockouts flowered earlier than the wild type (Chai *et al.*, 2012). Delayed heading may contribute favorably to higher yield through increased spikelet numbers and grain weight, the latter of which may benefit from a longer grain-filling period. In contrast, increased height or stature makes rice more susceptible to lodging, leading indirectly to yield loss. Thus, breeders who wish to employ this valuable yield-enhancing *O. rufipogon* allele may also need to use alleles at other loci that promote shorter stature, such as *sd-1,* which has an epistatic effect on the *APX9*^OR^ allele, as demonstrated by Kim *et al.* (2014).

The 3 bp insertion in the Hwaseong allele of *APX9* is responsible for the difference in DTH, PH, and grain size traits between Hwaseong and CR4379. It was interesting to find that all the *japonica* accessions included in this study had the 3 bp insertion, whereas *O. rufipogon* and *indica* accessions lacked this insertion. The finding that two *O. rufipogon* accessions, W1944 (Cai and Morishima, 2000) and W1943 (Huang *et al.*, 2012), putative ancestors of *japonica* rice, have the wild-type allele *APX9*^OR^ indicates that the 3 bp insertion occurred in *japonica* before the differentiation of tropical and temperate *japonica*, and that the insertion was subjected to artificial selection during the domestication of *japonica*. Because *APX9* underlies the QTL cluster affecting plant architecture and *APX9*^HS^ is associated with shorter stature, the PH trait might have undergone artificial selection during the domestication process and in modern breeding. The low genetic variation in genes surrounding *APX*^HS^ in *japonica* also indicates that this region was selected or common by descent in *japonica*. However, the possibility that selection acted on other genes in the region is not completely excluded. Considering that there are no reports or annotations for traits of agronomic importance in this region, except for *APX9* in the present study, *APX9* is likely the target for artificial selection. We also demonstrated that the 3 bp InDel in *APX9* clearly differentiates *japonica* and *O. rufipogon*. Collectively, our results indicate that the 3 bp insertion in *APX9* arose in a *japonica* ancestor in the early stages of *japonica* domestication, and the selection for this mutant allele was a critical step in the domestication process.

To date, numerous genes associated with crop performance traits such as heading date, grain weight, GL, and SPP have been cloned (reviewed in Bai *et al.*, 2012). It will be of great interest to understand how *APX9* interacts with alleles at other loci to generate transgressive variation for this valuable suite of agronomically important phenotypes.

## Supplementary data

The following supplementary data are available at *JXB* online.

Fig. S1. Measurement of antioxidant activity in Hwaseong, Dongjin, CR4379, and the transgenic lines.

Fig. S2. Expression pattern of *APX9* gene under drought stress in Hwaseong and CR4379, and in wild-type and transgenic plants.

Fig. S3. APX activity assay in Hwaseong, CR4379, and OE lines, and Dongjin and T-DNA insertional lines.

Fig. S4. Expression pattern of eight *APX* genes in Hwaseong, CR4379, and OE lines.

Fig. S5. Expression pattern of eight *APX* genes in Dongjin and four T-DNA insertion lines.

Fig. S6. Distribution of the 3 bp InDel of the *APX9* gene in 303 rice accessions.

Table S1. List of SSR and InDel markers used for fine mapping candidate genes and qRT–PCR primers.

Table S2. Rice accessions used in this study.

Table S3. TASUKE rice accessions used in this study.

Table S4. Comparison of agronomic traits among controls and transgenic lines at maturity.

Table S5. Comparison of agronomic traits between the control and *MS5* T-DNA lines at maturity.

Table S6. List of genes flanking *APX9*.

erab155_suppl_Supplementary_Figures_S1-S6

erab155_suppl_Supplementary_Tables_S1-S6

## Data Availability

All data supporting the findings of this study are available within the paper and within its supplementary data published online.
