# Supplementary material for: Natural variation in rice ascorbate peroxidase gene APX9 is associated with a yield-enhancing QTL cluster
Source: J Exp Bot. 2021 Apr 8;72(12):4254–68. doi: 10.1093/jxb/erab155 (PMC8163052; doi:10.1093/jxb/erab155)
Supplement: erab155_suppl_Supplementary_Figures_S1-S6 [file erab155_suppl_supplementary_figures_s1-s6.pdf]

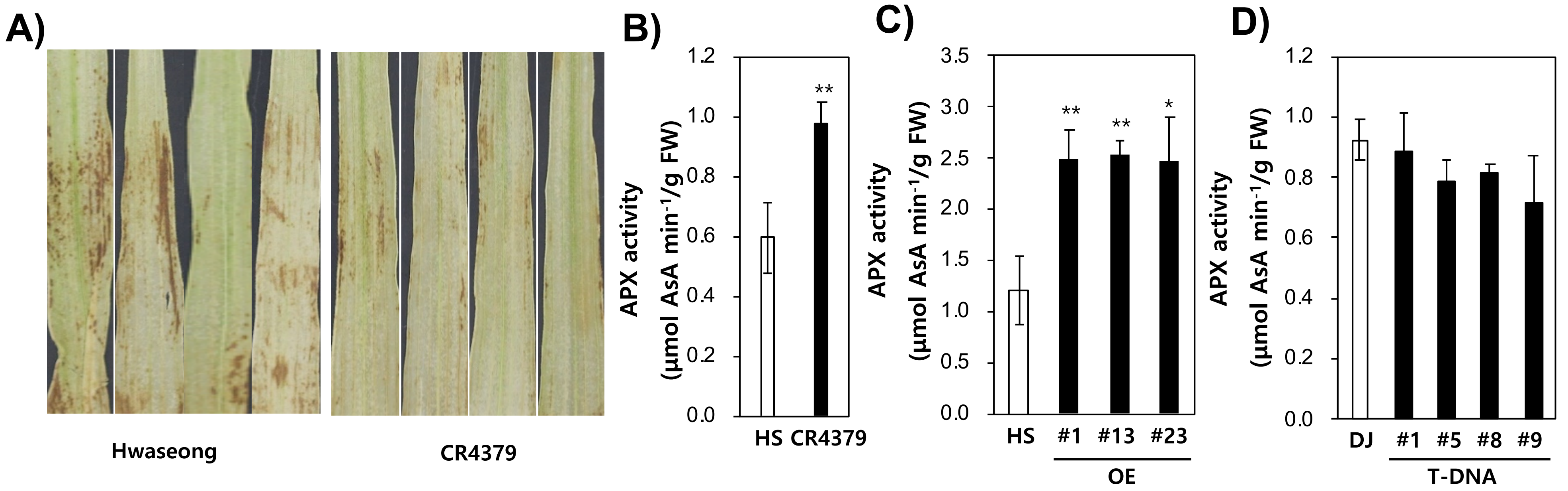

Fig.S1. Measurement of antioxidant activity in Hwaseong (HS) Dongjin (DJ), CR4379 and the transgenic lines. (A) *In situ* detection of H_2O_2 by DAB staining in HS and CR4379. (B, C, D) APX activity. Value represents mean ± SD calculated from three replicates. Asterisk indicates statistical significance between the control and each transgenic line by Student's *t*-test (* $P < 0.05$, ** $P < 0.01$, *** $P<0.001$).

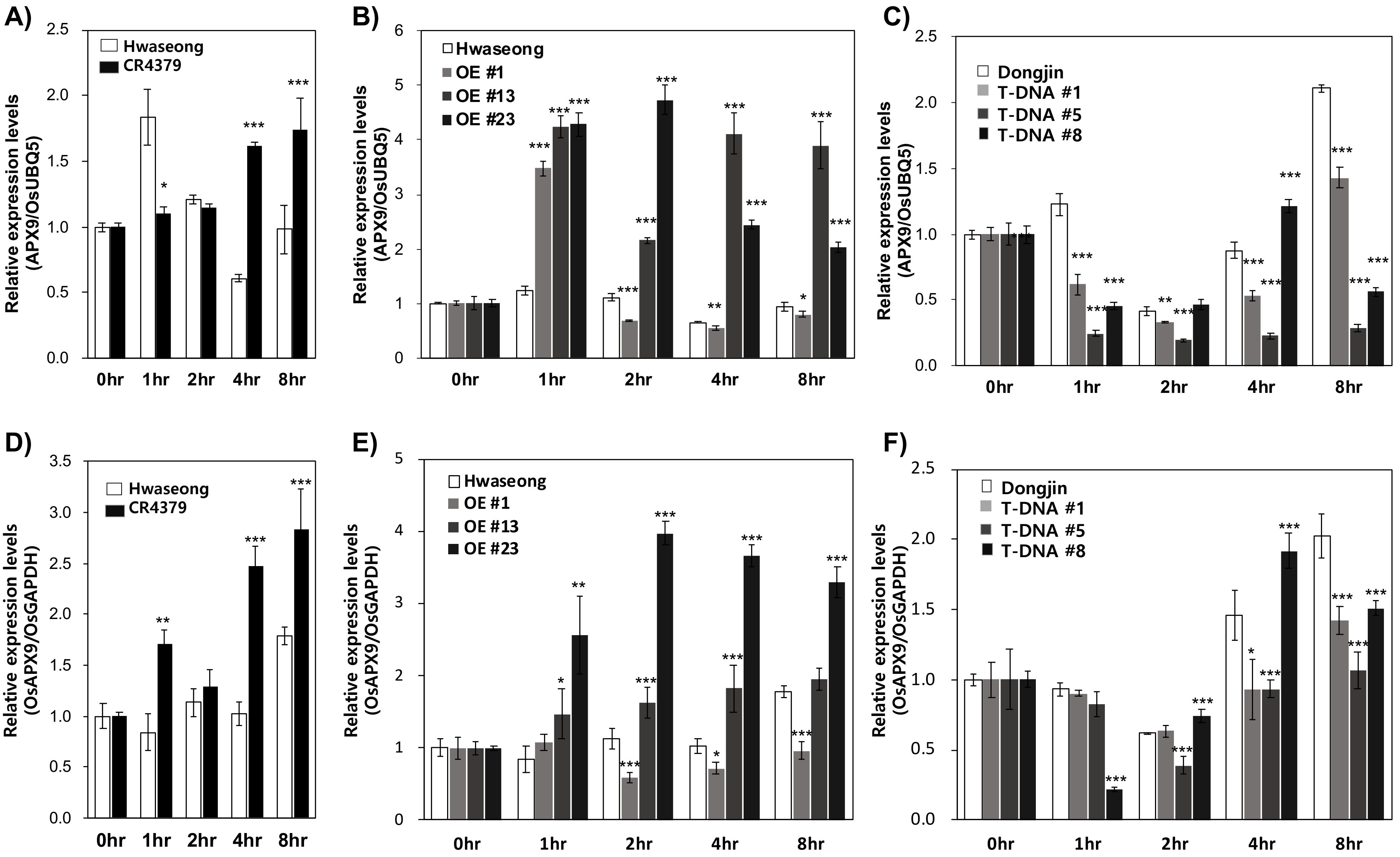

Fig. S2. Expression pattern of *APX9* gene under drought stress in Hwaseong and CR4379 (A, D) and in WT and transgenic plants (B, C, E, F). Relative expression levels were calculated based on the target gene/*OsUBQ5* (A, B, C) and /*OsGAPDH* (D, E, F).

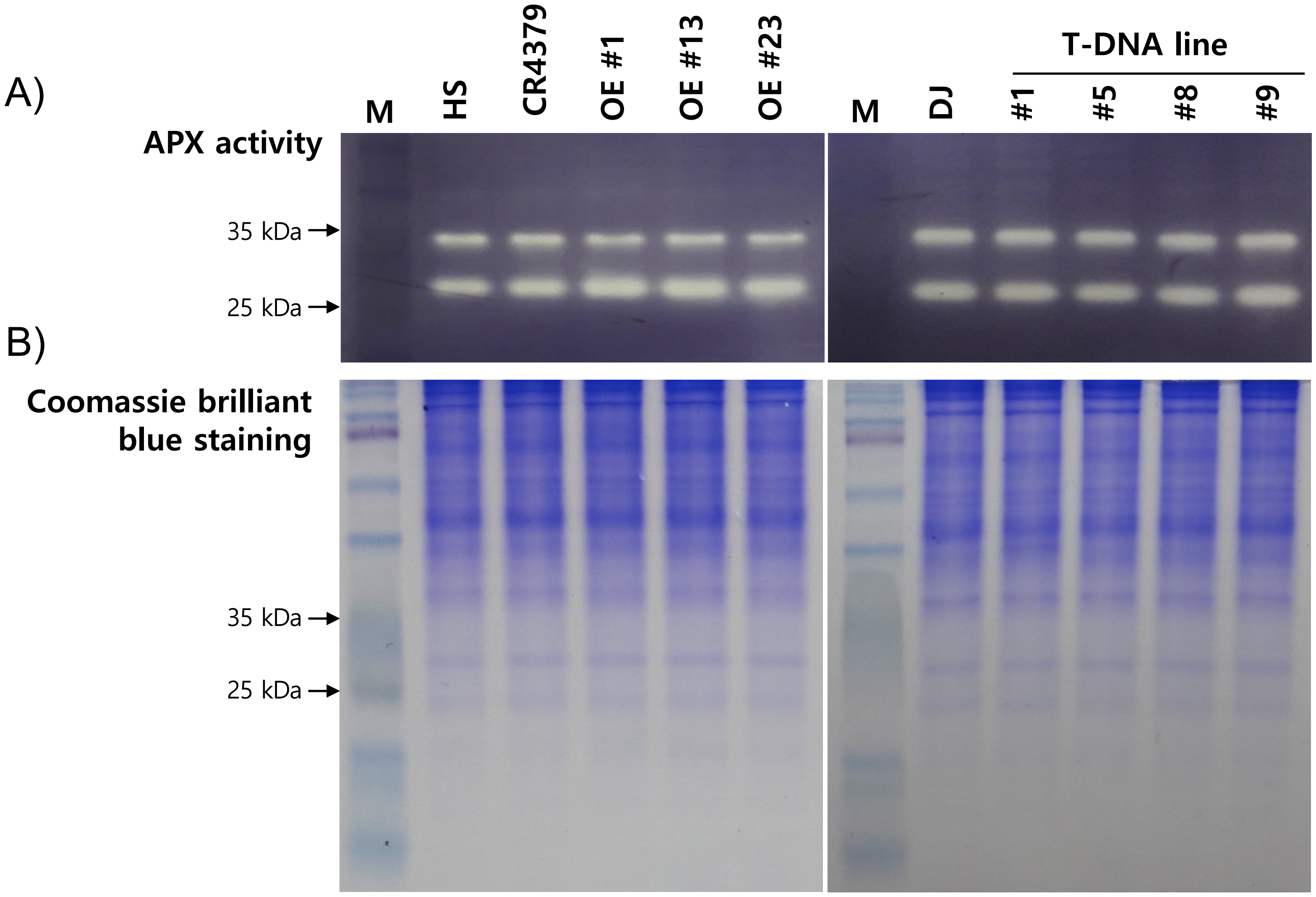

Fig.S3. APX activity assay in HS, CR4379 and OE lines (A, left) and DJ and T-DNA insertional lines (A, right). CR4379 and three OE lines showed more intensely-stained bands than Hwaseong at the expected molecular weight range of 25~35-kDa. B) Coomassie brilliant blue staining as a control.

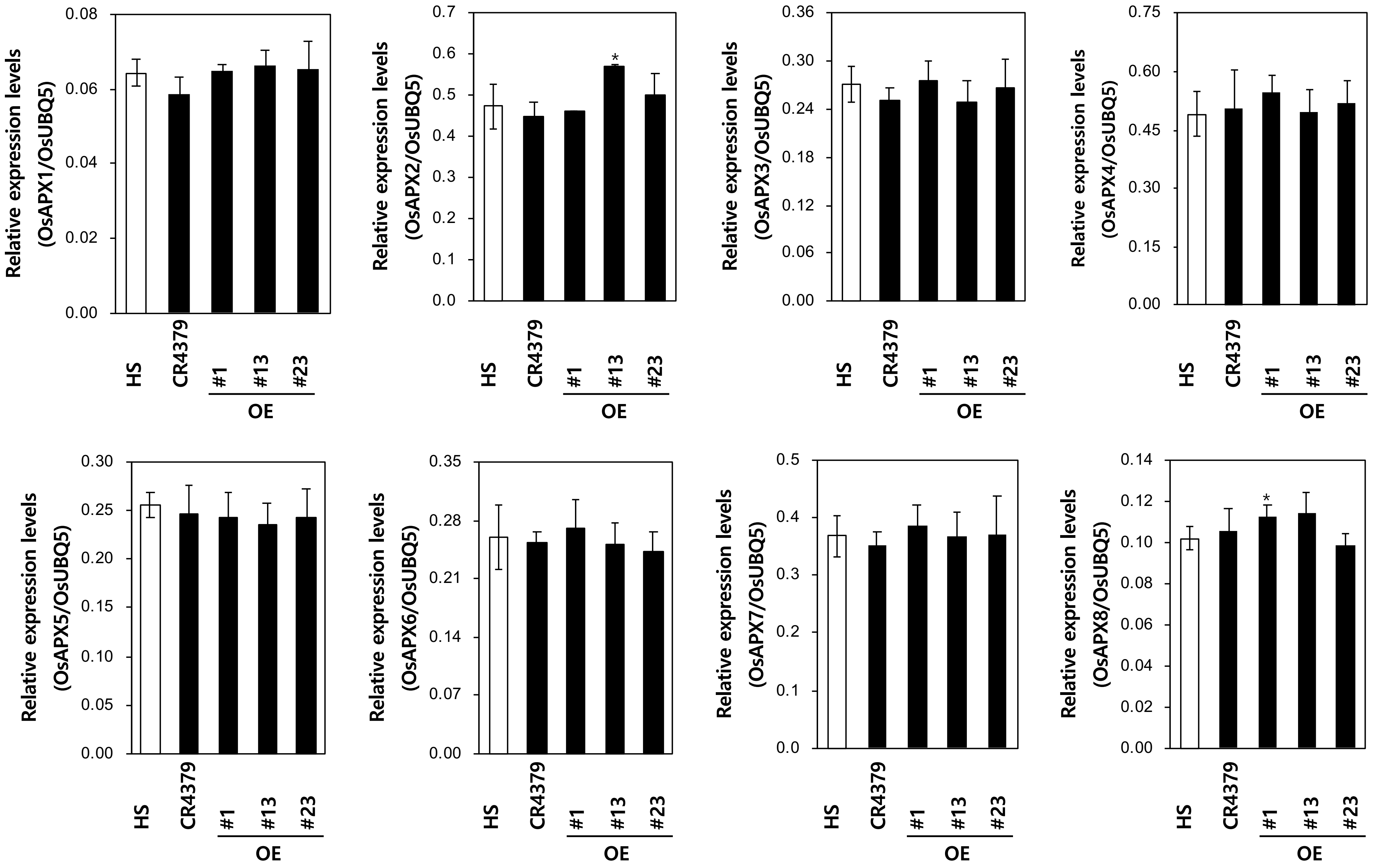

Fig.S4. Expression pattern of eight APX genes in Hwaseong (HS), CR4379 and OE lines. Relative expression levels were calculated based on the target gene/*OsUBQ5*.

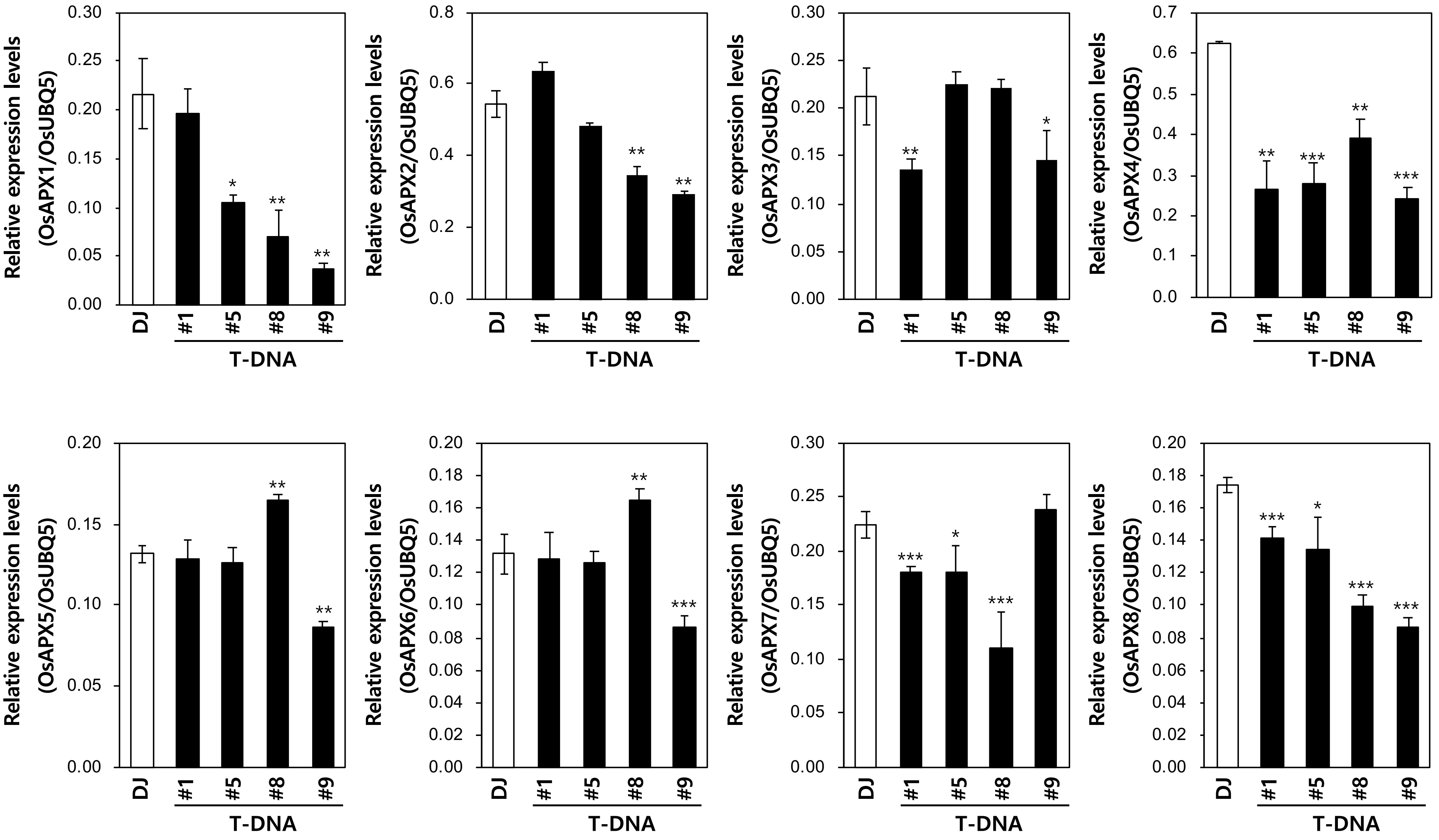

Fig.S5. Expression pattern of eight APX genes in Dongjin (DJ) and four T-DNA insertion lines. Relative expression levels were calculated based on the target gene/*OsUBQ5*.

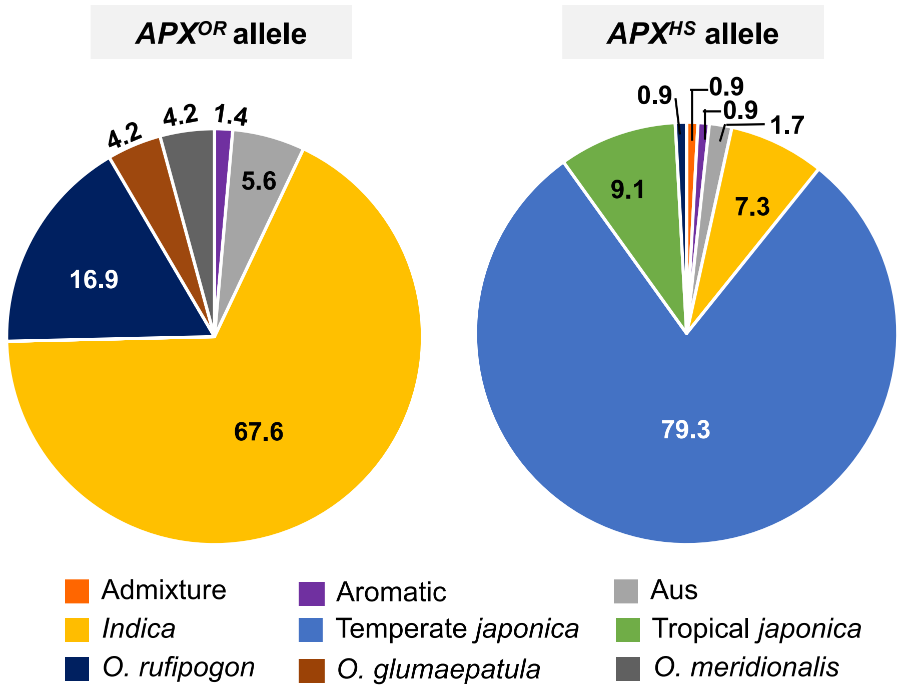

Ecotype	No. of accessions	***APX***^{OR} **allele**	***APX***^{HS} **allele**
Admixture	2	0	2
Aromatic	3	1	2
Aus	8	4	4
Indica	65	48	17
Temperate *japonica*	184	0	184
Tropical *japonica*	21	0	21
O. rufipogon	14	12	2
O. glumaepatula	3	3	0
O. meridionalis	3	3	0
**Total**	303	71	232

Fig.S6. Distribution of the 3-bp InDel of the *APX9* gene in 303 rice accessions. The *APX*^{OR} allele was not observed in admixture, temperate or tropical *japonica* accessions and was mainly found in both *indica* and *O. rufiipogon*.
